# Analysis of the Prognostic Value and Potential Molecular Mechanisms of TREM-1 Overexpression in Papillary Thyroid Cancer *via* Bioinformatics Methods

**DOI:** 10.3389/fendo.2021.646793

**Published:** 2021-05-27

**Authors:** Zhenyu Xie, Xin Li, Yuzhen He, Song Wu, Shiyue Wang, Jianjian Sun, Yuchen He, Yu Lun, Shijie Xin, Jian Zhang

**Affiliations:** Department of Vascular and Thyroid Surgery, The First Hospital, China Medical University, Shenyang, China

**Keywords:** TREM-1, papillary thyroid carcinoma, diagnostic biomarker, prognostic biomarker, immune infiltration, DNA methylation

## Abstract

**Background:**

Triggering receptor expressed on myeloid cells-1 (TREM-1) has been reported as a biomarker in many cancers. However, the biological function of TREM-1 in papillary thyroid carcinoma (PTC) remains unknown.

**Methods:**

We obtained TREM-1 expression data from The Cancer Genome Atlas (TCGA) database. Enrichment analysis of coexpressed genes and *TREM-1* methylation analysis were performed *via* LinkedOmics. The correlations between TREM-1 and immune infiltrates were investigated *via* ESTIMATE, TIMER and TISIDB. We analyzed the association of TREM-1 expression with pan-cancer overall survival *via* Gene Expression Profiling Interactive Analysis (GEPIA).

**Results:**

*TREM-1* has lower methylation levels and higher expression levels in PTC tissues compared to normal tissues. TREM-1 expression is significantly associated with poor prognosis, advanced T classification, advanced N classification, and an increased incidence of *BRCA2* and *BRAF* mutations. Genes coexpressed with *TREM-1* primarily participate in immune-related pathways. TREM-1 expression is positively correlated with immune infiltration, tumor progression and poor overall survival across cancers.

**Conclusions:**

TREM-1 is a good prognostic and diagnostic biomarker in PTC. TREM-1 may promote thyroid cancer progression through immune-related pathways. Methylation may act as an upstream regulator of TREM-1 expression and biological function. Additionally, TREM-1 has broad prognostic value in a pan-cancer cohort.

## Introduction

Thyroid carcinoma (1) is the most prevalent endocrine malignancy, and its global incidence has rapidly increased in recent decades (2). Papillary thyroid carcinoma (PTC) is the most common type of TC and accounts for nearly 85% of all thyroid cancers (3). With standardized treatment, PTC often exhibits a favorable prognosis, with a 10-year survival rate of 93% (4). However, a high tendency of early lymph node metastasis or recurrence occurs in more than 30% cases (5). Therefore, identifying reliable and accurate biomarkers for the diagnosis and prognosis of PTC is essential.

Triggering receptor expressed on myeloid cells-1 (TREM-1) is a cell surface receptor belonging to the immunoglobulin superfamily (6). The function of TREM-1 is to enhance the inflammatory response by inducing large amounts of proinflammatory mediators (6). Human TREM-1 is predominantly expressed by blood neutrophils and by a subset of monocytes/macrophages (7). Furthermore, TREM-1 is also highly expressed by neutrophils and epithelial cells in skin and lymph nodes infected with bacteria or fungi (8). The tissue distribution of TREM-1 expression indicates a role for TREM-1 in inflammation (9). Because TREM-1 is widely distributed throughout the body and plays an important role in the inflammatory process, it has attracted great attention in the context of tumor formation, development and metastasis. TREM-1 overexpression has been reported in many malignancies and correlates with poor prognosis. A previous study has indicated that lung cancer cells upregulate TREM-1 expression and that tumor-associated macrophages have increased levels of TREM-1 (10). Functional experiments involving hepatocellular carcinoma (HCC) have suggested that TREM-1 promotes proliferation, increases invasiveness and inhibits apoptosis of HCC cells (11, 12). TREM-1 is involved in the establishment of a prognossis model for liver cancer (13). High-risk prognostic subgroups defined by TREM-1 expression have been identified for colorectal cancer (CRC) (14). In addition, signaling *via* TREM-1 is significantly related to the development and progression of chronic lymphocytic leukemia (15). TREM-1 is induced in macrophages by the androgen receptor signaling pathway in prostate cancer, and increases the motility and invasive capacity of prostate cancer cells (16). In recent years, many studies have suggested various strategies for regulating TREM-1 expression and activity to block the interaction between TREM-1 and its ligands to treat a number of diseases, including cancer (17, 18).

However, the biological function of TREM-1 in PTC remains unknown. Here, we investigated TREM-1 expression in PTC patients using data from The Cancer Genome Atlas (TCGA) and the National Center for Biotechnology Information Gene Expression Omnibus (GEO) databases. Through multidimensional analysis, we evaluated the genomic functional networks related to TREM-1 in PTC and further explored the role of TREM-1 in tumor immunity. Our results suggested that TREM-1 may be a new potential target for PTC diagnosis and treatment.

## Materials and Methods

### Materials

The TCGA THCA dataset, containing normal thyroid samples (N) =58 and PTC samples (T) =512, was selected as the discovery cohort. The normalized RNA-seq data (FPKM, level three), DNA methylation data and single nucleotide variation data (VarScan) were downloaded from TCGA *via* the Genomic Data Commons (GDC) portal (https://portal.gdc.cancer.gov/). The clinical data of the TCGA THCA dataset were downloaded from the University of California at Santa Cruz (UCSC) Xena browser (https://xena.ucsc.edu/).

To assess the diagnostic value of TREM-1, we selected data-sets containing both PTC and normal tissues in the GEO database. The following six RNA-seq microarray datasets were downloaded from the National Center for Biotechnology Information GEO database (http://www.ncbi.nlm.nih.gov/geo): GSE3467 (N=9, T=9), GSE27155 (N=4, T=51), GSE33630 (N=45, T=49), GSE53157 (N=3, T=15), GSE58545 (N=18, T=27) and GSE60542 (N=30, T=33).

### Comparison of TREM-1 Expression Between PTC and Normal Thyroid Tissues

TREM-1 mRNA expression in PTC and normal thyroid tissues was compared based on TCGA data. We defined the top quartile based on the mRNA expression rank in the THCA dataset as the H-TREM-1 group, and the remaining samples were defined as the L-TREM-1 group. Receiver operating characteristic (ROC) curve analysis was used to evaluate the diagnostic efficacy of TREM-1. The diagnostic value of TREM-1 was further verified in 6 GEO datasets (GSE3467, GSE27155, GSE33630, GSE53157, GSE58545 and GSE60542).

Additionally, direct comparison of TREM-1 protein expression between PTC and normal thyroid tissues was performed by using immunohistochemical images from the Human Protein Atlas (https://www.proteinatlas.org).

### Analysis of the Potential Mechanism of TREM-1 in PTC

#### LinkedOmics Database Analysis

LinkedOmics is an online website for analyzing multiomics data within and across 32 cancer types (http://www.linkedomics.org/) (19). Pearson correlation coefficient values were used to analyze TREM-1 coexpression, and the results were visualized with a volcano plot and heatmap. The functional modules of LinkedOmics were based on the RNA-seq and methylation data of TREM-1. In addition, gene set enrichment analysis (GSEA) was performed for gene ontology (GO) biological process terms and Kyoto Encyclopedia of Genes and Genomes (KEGG) pathways. We used a weighted set cover approach for redundancy reduction, and we set the minimum number of genes to 10 and the number of simulations to 1000. In addition, we investigated the relationships between TREM-1 expression and gene mutations in PTC as well as between TREM-1 methylation and clinical parameters.

#### Protein-protein Interaction (PPI) Network Analysis

The 100 genes with the most significant coexpression with TREM-1 in LinkedOmics were input into String (version 11.0, https://string-db.org/) to analyze the interaction network of the encoded proteins. The minimum required interaction score was 0.7 (high confidence), and the results were visualized with Cytoscape (v3.7.2).

### Analysis of the Relationship Between TREM-1 and Immune Infiltration in PTC

#### ESTIMATE

Using ESTIMATE (20)to evaluate the immune cell infiltration level (immune score), matrix content (stromal score), comprehensive score (ESTIMATE score) and tumor purity of each THCA sample, we compared the tumor microenvironment among patients with different TREM-1 expression levels.

#### TIMER

The TIMER online database (21) was used to analyze and visualize the associations between TREM-1 and 6 subtypes of tumor-infiltrating immune cells [B cells, CD4+ T cells, CD8+T cells, macrophages, neutrophils and dendritic cells (DCs)]. Purity-corrected partial Spearman-correlation coefficients were calculated.

#### TISIDB

We used TISIDB, a web portal for analyzing tumor and immune system interactions (22), to analyze the Spearman correlation between TREM-1 expression and 28 types of tumor-infiltrating lymphocytes (TILs), immunomodulators (immunoinhibitors, immunostimulators and MHCs), chemokines and receptors as well as tumor stage and overall survival (OS) across human cancers.

#### CIBERSORT

CIBERSORT (23) is a deconvolution algorithm that uses RNA-seq data to calculate the composition ratio of 22 immune cells in each blood or tissue sample. We performed 1,000 permutations and kept samples with *p <*0.05 to ensure the accuracy of the results, and the sum of various immune cells was 1. In this study, CIBERSORT was used to analyze the Spearman correlation between TREM-1 expression and the proportion of various immune cells in THCA.

#### Immune Cell Abundance Identifier (ImmuCellAI)

ImmuCellAI (24) (http://bioinfo.life.hust.edu.cn/web/ImmuCellAI/) is a method based on single sample gene set enrichment analysis (ssGSEA), which can be used to accurately estimate the abundance of 24 immune cells from gene expression data, including 18 T cell subgroups. We used ImmuCellAI to analyze the Spearman correlation between TREM-1 expression and the abundance of various immune cells in THCA.

### Analysis of TREM-1 Methylation in PTC

Based on DNA methylation data downloaded from TCGA, we compared the methylation levels of TREM-1 between PTC and normal tissues. In addition, we performed Spearman correlation analysis to assess the correlations between TREM-1 expression and methylation at each methylation site in the TREM-1 gene. The remaining methylation analysis was performed by LinkedOmics.

### Gene Expression Profiling Interactive Analysis (GEPIA)

We used the online pan-cancer analysis platform, GEPIA (http://gepia.cancer-pku.cn/) (25), to analyze the differences in TREM-1 expression levels between tumors and normal tissues across human cancers (P-value <0.05 and |logFC| >1) and the association of TREM-1 expression with pan-cancer OS.

### Statistical Analysis

The Chi-square test was used to assess differences in clinical parameters between the L-TREM-1 and H-TREM-1 groups. The Spearman method was used to test correlations. The Mann-Whitney test was used to compare data between two groups. The log-rank method was used to calculate significant P values in the survival analysis. The R software (v3.6.0) and SPSS software (v25.0) were used for statistical processing. Data visualization was performed with GraphPad Prism V.8.0 and R software.

## Results

### Diagnostic Value of TREM-1 in PTC

**Figure 1:**
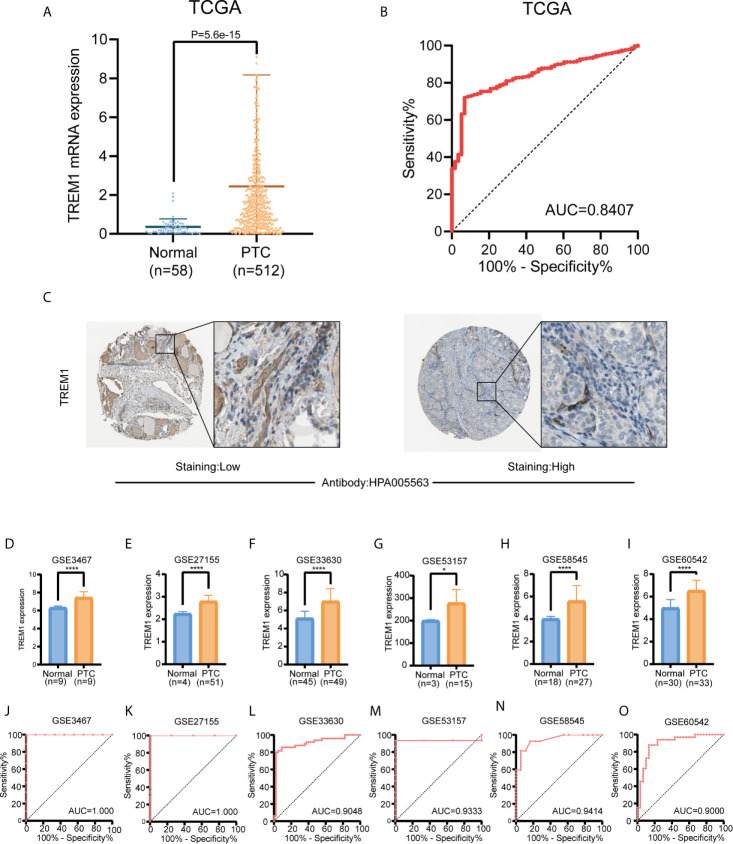
Diagnostic value of TREM-1 in PTC. **(A)** Comparison of TREM-1 mRNA expression between PTC and normal tissues in TCGA. **(B)** Diagnostic efficacy of TREM-1 as shown by the ROC curve. **(C)** Comparison of immunohistochemical images indicating TREM-1 expression in PTC and normal thyroid tissues. **(D–I)** TREM-1 mRNA expression in the six GEO verification cohorts (GSE3467, GSE27155, GSE33630, GSE58545, GSE53157 and GSE60542). **(J–O)** ROC curve showing the diagnostic performance of TREM-1 expression in the verification cohorts. *P < 0.05, ****P < 0.0001 here.

We initially evaluated TREM-1 mRNA levels in PTC tissues from TCGA. Comparison of normal thyroid tissues (n=58) and PTC tissues (n=512) revealed that TREM-1 mRNA expression was significantly higher in PTC tissues than in normal tissues (P<0.0001) (**Figure 1A**). ROC curve analysis was performed to evaluate the diagnostic value of TREM-1 (**Figure 1B**). Additionally, the immunohistochemical results indicated that TREM-1 protein expression was much higher in PTC tissues than in normal tissues (**Figure 1C**). Furthermore, we analyzed gene expression data from 6 GEO cohorts, and all results supported the above conclusion [GSE3467 (P<0.0001), GSE27155 (P<0.0001), GSE33630 (P<0.0001), GSE58545 (P<0.05), GSE53157 (P<0.0001) and GSE60542 (P<0.0001)] (**Figures 1D–I**). ROC curve analysis was performed to evaluate the diagnostic value of TREM-1 using data from GEO cohorts (**Figures 1J–O**). The areas under the ROC curve (AUCs) were 0.8407, 1.000, 1.000, 0.9048, 0.9333, 0.9414, and 0.9000 in the seven cohorts, suggesting that TREM-1 is a potential biomarker for distinguishing PTC cases from normal controls.

### Prognostic Value of TREM-1 in PTC

Univariate and multivariate analyses of TCGA cohort were conducted to assess the prognostic value of TREM-1 (**Table 1**). Univariate analysis revealed that age (per year of age), clinical stage, tumor mutation burden (TMB) and TREM-1 expression are significant risk factors for progression-free survival (PFS) (P<0.001). Multivariate analysis further revealed that high TREM-1 expression is an independent prognostic biomarker for poor PFS [hazard ratio (HR) = -1.032; 95% CI 1.012-1.053].

**Table 1:** Results of univariate and multivariate logistic regression analysis.

Variables	Univariate analysis		Multivariate analysis	
	Hazard ratio (95%CI)	P value	Hazard ratio (95% CI)	P value
Age	1.035(1.017−1.053)	<0.001	1.014(0.990−1.039)	0.267
Sex	1.398(0.771−2.536)	0.27	1.232(0.663−2.290)	0.509
Clinical stage	1.739(1.367−2.212)	<0.001	1.429(1.048−1.948)	0.024
TMB	2.963(1.636−5.367)	<0.001	1.388(0.633−3.043)	0.412
TREM-1	1.042(1.024−1.061)	<0.001	1.032(1.012−1.053)	0.001

### Relationship of TREM-1 Expression With Clinical Parameters

The PTC patients in TCGA cohort were separated into two groups according to TREM-1 expression level (H-TREM-1 and L-TREM-1). Comparison of the two groups indicated significant differences in the N and T classification between the groups (P<0.01). In addition, the H-TREM-1 group included more patients with classical and tall cell types but fewer patients with follicular type cells (P<0.01). Notably, high TREM-1 expression was significantly associated with lymph node metastasis and advanced T classification (**Table 2**).

**Table 2:** Association between TREM-1expression and clinical parameters.

Clinical parameter	L-TREM-1 (n=376, %)	H-TREM-1 (n=125, %)	P value
Age (y)			
<55	248 (66.0)	86 (68.8)	0.559
≥55	128 (34.0)	39 (31.2)	
Sex			
Female	281 (74.7)	85 (68.0)	0.142
Male	95 (25.3)	40 (32.0)	
Clinical stage			
I	219 (58.6)	62 (49.6)	0.06
II	42 (11.2)	10 (8.0)	
III	73 (19.5)	38 (30.4)	
IV	40 (10.7)	15 (12.0)	
NA			
Metastasis stage			
M0	209 (97.2)	73 (96.1)	0.617
M1	6 (2.8)	3 (3.9)	
NA	161	49	
N stage			
N0	186 (55.2)	43 (37.7)	0.001
N1	151 (44.8)	71 (62.3)	
NA	39	11	
T stage			
T1	117 (31.2)	25 (20.2)	0.003
T2	130 (34.7)	34 (27.4)	
T3	112 (29.9)	58 (46.8)	
T4	16 (4.3)	7 (5.6)	
NA	1	1	
Pathologic type			
Classical	260 (69.1)	95 (76.0)	<0.001
Follicular	89 (23.7)	12 (9.6)	
Tall cell variant	19 (5.1)	17 (13.6)	
Other	8 (2.1)	1 (0.8)	
BRAF			
Wild-type	149 (41.5)	46 (37.7)	0.46
Mutated	210 (58.5)	76 (62.3)	
NA	17	3	
RAS			
Wild-type	310 (86.4)	111 (91.0)	0.181
Mutated	49 (13.6)	11 (9.0)	
NA	17	3	

### TREM-1-Related Genes in PTC

**Figure 2:**
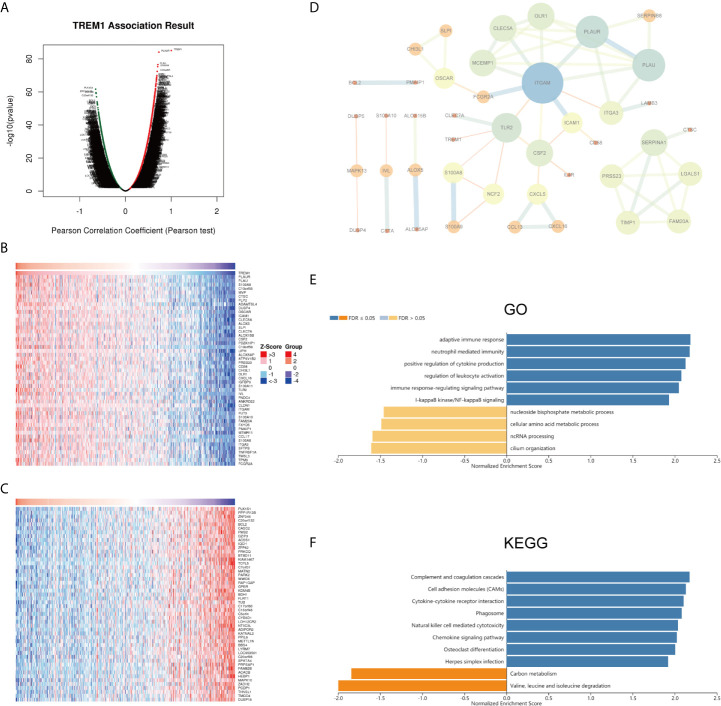
Potential mechanisms of TREM-1 in PTC. **(A)** Genes highly correlated with TREM-1 identified by Pearson correlation analysis in the THCA cohort. **(B)** Heatmaps showing the top 50 genes positively and **(C)** negatively correlated with TREM-1 in the TCHA cohort. **(D)** PPI of the top 100 significantly correlated genes. **(E)** GO biological process terms and **(F)** KEGG pathways significantly enriched in genes coexpressed with TREM-1 in the THCA cohort.

To gain insights into the biological meaning of TREM-1 in PTC, the functional module in LinkedOmics was used to examine the TREM-1 coexpression mode in the THCA cohort. A total of 9616 genes showed significant positive correlations with TREM-1, whereas 10312 genes showed significant negative correlations [false discovery rate (FDR) < 0.01] (**Figure 2A**). The top 50 genes with the most significant positive and negative correlations with TREM-1 are shown in the heat map, respectively (**Figures 2B, C**). Moreover, we constructed a PPI network with 43 hub genes and found that ITGAM plays a key role (**Figure 2D**). Regarding the relationship between TREM-1 and mutations in other genes, mutations in BRCA2 and BRAF were most closely related to TREM-1 (**Figure S1A**). Furthermore, TREM-1 expression levels in the BRAF mutant group were significantly higher than that in the BRAF wild-type group (**Figure S1B**).

### Functional Enrichment Analysis of TREM-1

Annotation of significantly enriched GO terms by GSEA showed that genes coexpressed with TREM-1 were primarily involved in functions described by the following terms: adaptive immune response; neutrophil-mediated immunity; positive regulation of leukocyte activation; regulation of leukocyte activation; immune response-regulating signaling pathway; and I-kappaB kinase/NF-kappaB signaling. In contrast, activities related to the following terms were inhibited: nucleoside bisphosphate metabolic process; cellular amino acid metabolic process; ncRNA processing; and cilium organization (**Figure 2E**). KEGG pathway analysis showed enrichment of these genes in the following pathways: complement and coagulation cascades; cell adhesion molecules (CAMs); cytokine-cytokine receptor interaction; phagosome; natural killer cell mediated cytotoxicity; and chemokine signaling pathway (**Figure 2F**).

### Relationship of TREM-1 Expression With Immune Infiltration

**Figure 3:**
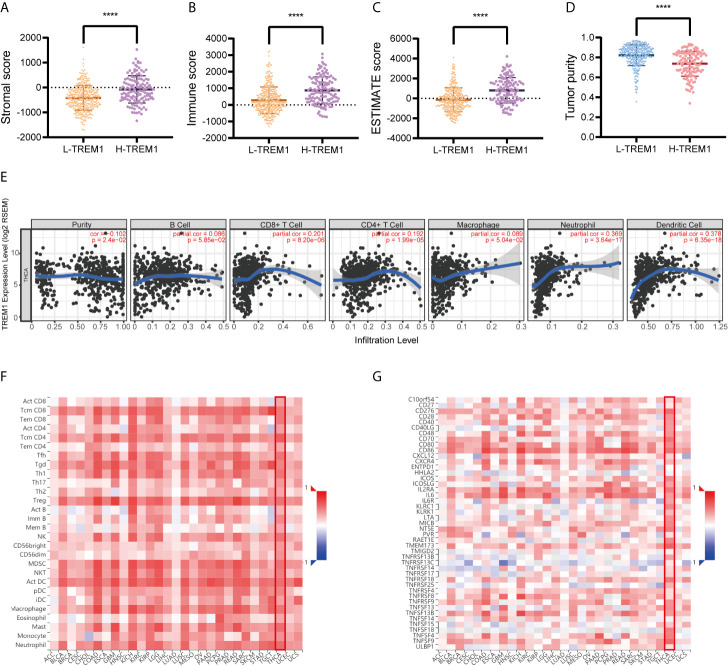
TREM-1 is closely related to immunity in PTC. ESTIMATE analysis of **(A)** stromal scores, **(B)** immune scores, **(C)** ESTIMATE scores and **(D)** tumor purity between the L-TREM-1 and H-TREM-1 groups. **(E)** TIMER analysis of purity-corrected partial Spearman correlations between the expression of TREM-1 and the infiltration of 6 types of immune cells in the THCA cohort. **(F)** Correlation analysis between the expression of TREM-1 and 28 types of TILs across human cancers *via* TISIDB. **(G)** Correlation analysis between the expression of TREM-1 and the levels of immunostimulators across human cancers *via* TISIDB. ****P < 0.0001 here.

The stromal score, immune score, ESTIMATE score and tumor purity data for TCGA cohort were obtained and compared between the H-TREM-1 and L-TREM-1 groups (**Figures 3A–D**). The H-TREM-1 group had a higher stromal score (P<0.0001) and ESTIMATE score but a lower tumor score (P<0.0001) and tumor purity (P<0.0001). Correlation analysis of TREM-1 expression with tumor purity revealed that TREM-1 may influence the immune status of the tumor microenvironment. Moreover, we investigated whether TREM-1 expression is correlated with immune infiltration in PTC *via* the TIMER database. TREM-1 expression was significantly correlated with tumor purity (r=-0.102, P<0.05) and the infiltration levels of the dominant immune cells (**Figure 3E**). Specifically, TREM-1 expression was significantly correlated with the infiltration levels of dendritic cells, neutrophils and CD8+T cells. In addition, TISIDB analysis suggested that TREM-1 expression was positively correlated with the levels of 28 TIL types (**Figure 3F**) and immunostimulators (**Figure 3G**) across human cancers and that these correlations were particularly significant in THCA. In addition, correlations between TREM-1 expression and the levels of immunoinhibitors, MHCs, chemokines and receptors were analyzed (**Figure S2**).

### Relationship of TREM-1 Expression With Various Immune Cells

**Figure 4:**
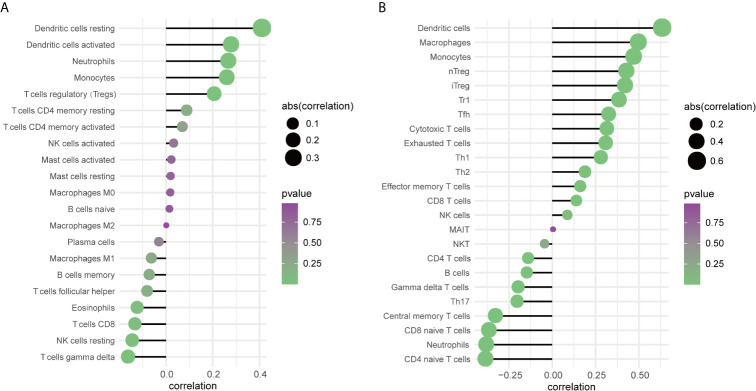
The relationship between TREM-1 and immune cells in PTC. **(A)** Correlation analysis between TREM-1 expression and the proportion of immune cells in PTC (CIBERSORT, n=259). **(B)** Correlation analysis between TREM-1 expression and the abundance of immune cells in PTC (ImmuCellAI, n=512).

To further explore the relationship between TREM-1 expression levels and the immune system, we used CIBERSORT to examine the relationship between TREM-1 expression levels and the proportion of different immune cells in PTC tissue, as well as ImmuCellAI to examine the relationship between TREM-1 expression levels and various immune cell abundances in PTC tissues. The findings (**Figure 4**) revealed that TREM-1 over-expression was related to a higher proportion of resting dendritic cells, activated dendritic cells, neutrophils, monocytes, Tregs and resting memory CD4+ T cells (P<0.05), while TREM-1 over expression is related to a decline in the proportion of T cells gamma delta, resting NK cells, CD8+ T cells, and eosinophils (P<0.05) (**Table S1**). TREM-1 over expression was also associated with a higher abundance of dendritic cells, macrophages, monocytes, nTregs, iTregs, Tr1, Tfh, cytotoxic T cells, exhausted T cells, Th1, Th2, effector memory T cells and CD8+ T cells (P<0.05) and a lower abundance of naive CD4+ T cells, neutrophils, naive CD8+ T cells, central memory T cells, Th17, T cells gamma delta, B cells and CD4+ T cells (P<0.05) (**Table S2**). The results reflect that TREM-1 expression has a stronger correlation with innate immune cells.

### DNA Methylation of TREM-1

**Figure 5:**
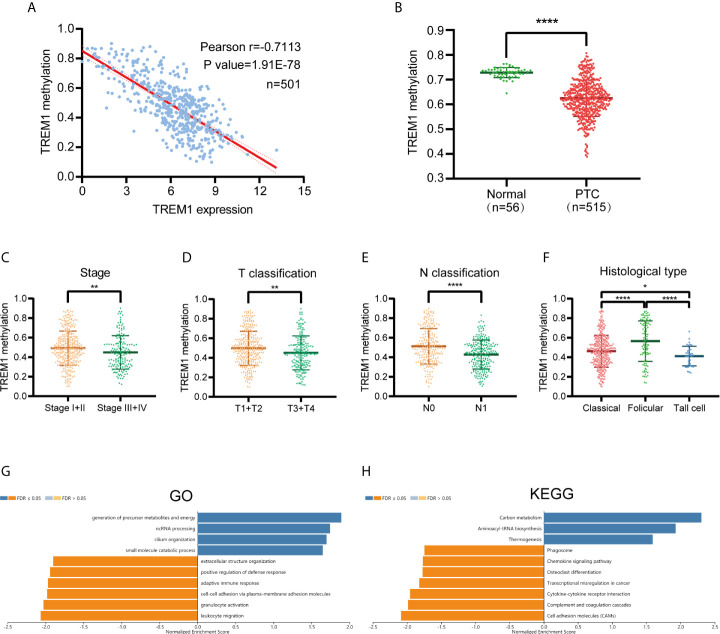
TREM-1 Methylation in PTC. **(A)** Correlation analysis between TREM-1 methylation and TREM-1 expression in PTC **(B)** TREM-1 methylation levels were compared between PTC and normal tissues. TREM-1 methylation level among patients with different **(C)** clinical stages, **(D)** T classifications, **(E)** N classifications and **(F)** histological types of PTC. **(G)** GO biological process terms and **(H)** KEGG pathways significantly altered by TREM-1 methylation in the THCA cohort. *P < 0.05, **P < 0.01 and ****P < 0.0001 here.

We performed Pearson correlation analysis to assess the correlation between TREM-1 expression and TREM-1 DNA methylation based on DNA methylation data from patients with PTC downloaded from TCGA (**Figure 5A**). The methylation level of TREM-1 DNA was significantly negatively correlated with TREM-1 mRNA expression levels (r=-0.7113, P<0.0001). Methylation at DNA TREM-1 methylation sites, including cg21328082, cg10981439, cg09310966, cg06196379, c18505453, cg03843170, cg04451353 and cg17430214, exhibited the most significant negative correlations with TREM-1 expression levels (**Table 3**). Additionally, the methylation level of TREM-1 in PTC tissues was significantly lower than in normal tissues (P<0.0001) (**Figure 5B**). Therefore, we hypothesized that DNA methylation may be the upstream mechanism regulating TREM-1 in PTC. Patients were classified according to clinical indicators, and we compared their TREM-1 methylation levels (**Figures 5C–F**). Lower methylation levels of TREM-1 were found in patients with advanced clinical stage, advanced T classification, advanced N classification and tall cell variant PTC. To understand the biological significance of TREM-1 methylation in THCA, a functional module of LinkedOmics was used to examine TREM-1 coexpression patterns in the THCA cohort. Using RNAseq, we screened 19928 genes related to TREM-1 methylation (false discovery rate (FDR) <0.01). The GO (biological process) analysis results derived by GSEA were significant. The results indicated that TREM-1 methylation-coexpressed genes primarily participate in generation of precursor metabolites and energy, ncRNA processing, cilium organization, and small molecule catabolic process, while extracellular structure organization, positive regulation of defense response, adaptive immune response, cell-cell adhesion *via* plasma-membrane adhesion molecules, granulocyte activation and leukocyte migration were inhibited (**Figure 5G**). KEGG pathway analysis showed that genes related to carbon metabolism, aminoacyl-tRNA biosynthesis, and thermogenesis were activated, while phagosome, chemokine signaling pathway, osteoclast differentiation, transcriptional misregulation in cancer, cytokine-cytokine receptor interaction, complement and coagulation cascades and cell adhesion molecules (CAMs) were inhibited (**Figure 5H**).

**Table 3:** Spearman correlation between TREM-1 methylation sites and TREM-1 expression.

Methylation site	Spearman r	P value
cg21328082	-0.679	3.49E-69
cg10981439	-0.581	1.13E-46
cg09310966	-0.447	5.07E-26
cg06196379	-0.41	9.42E-22
cg18505453	-0.289	4.16E-11
cg03843170	-0.188	2.14E-05
cg04451353	-0.105	1.90E-02
cg17430214	NA	NA

NA, Not applicable.

### Generalization Value of TREM-1 Across Cancers

**Figure 6:**
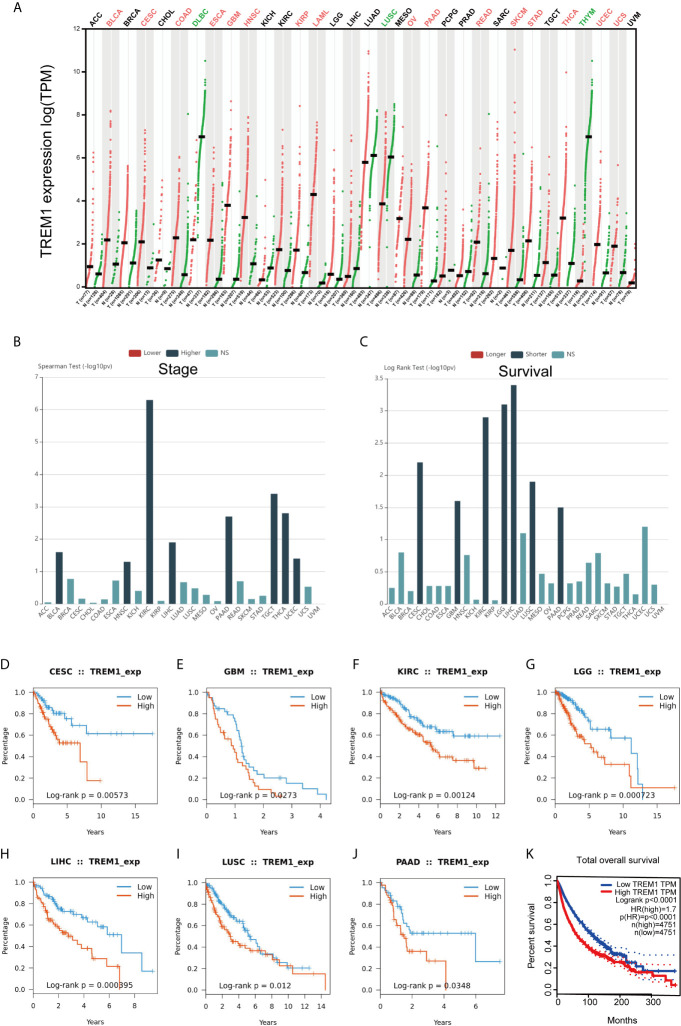
Generalization value of TREM-1 across cancers. **(A)** Comparison of TREM-1 mRNA expression between cancer and paracancerous tissues across cancers. **(B)** Associations between TREM-1 expression and stage across human cancers. **(C)** Associations between TREM-1 expression and OS across human cancers. K-M survival analysis between the L-TREM-1 and H-TREM-1 groups in the **(D)** CESC, **(E)** GBM, **(F)** KIRC, **(G)** LGG, **(H)** LIHC, **(I)** LUSC, **(J)** PAAD and **(K)** pan-cancer cohorts.

To investigate whether TREM-1 has broad value across cancers, we performed a series of studies on TREM-1 in a pan-cancer cohort. GEPIA showed that the TREM-1 expression status varied across cancers (**Figure 6A**). TISIDB analysis showed that high expression of TREM-1 in the pan-cancer cohort was accompanied by a more advanced tumor stage (**Figure 6B**) and shorter OS time (**Figure 6C**). Kaplan-Meier (K-M) survival analysis showed that the high TREM-1 groups in the cervical squamous cell carcinoma and endocervical adenocarcinoma (CESC), glioblastoma multiforme (GBM), kidney renal clear cell carcinoma (KIRC), brain lower grade glioma (LGG), liver hepatocellular carcinoma (LIHC), lung squamous cell carcinoma (LUSC) and pancreatic adenocarcinoma (PAAD) cohorts had significantly shorter OS times (**Figures 6D–J**). The difference was the most significant in the survival statistics of a large sample in the pan-cancer cohort (N = 9502, HR = 1.7, P <0.0001) (**Figure 6K**).

## Discussion

The present study utilized PTC patient sequencing data in public databases and presented the first report of the expression level and biological function of TREM-1 in PTC. The expression level of TREM-1 in PTC tissues was significantly higher than that in noncancerous thyroid tissues at both the transcriptional and translational levels. Furthermore, our results indicated the prognostic and diagnostic value of TREM-1. Our findings suggest a clinical correlation of disease severity and subclassification with gene expression levels, indicating that TREM-1 may affect disease progression. Previous research has identified a significant correlation between TREM-1 mRNA expression and TREM-1 DNA methylation levels (26). Our TREM-1 methylation analysis data also implied that DNA methylation is an epigenetic regulatory mechanism of TREM-1 resulting in transcriptional silencing. The finding of TREM-1 hypomethylation also validated its high protein levels in PTC and its effect on disease progression. Our results suggested that the functional consequences of TREM-1 mainly include effects on immune-related responses. Therefore, we adopted the ESTIMATE and TIMER algorithms to investigate the infiltration of immune cells and found that TREM-1 expression is associated with tumor-infiltrating immune cells, and exhibited positive associations with immune infiltration of dendritic cells, neutrophils and CD8+T cells. TREM-1 has extensive prognostic value in pan-cancer.

TREM-1 is expressed at late stages of myeloid cell differentiation (27) and is found on monocytes/macrophages and neutrophils with high CD14 expression (7). TREM-1 acts as an important mediator of intracellular signaling by immune cells during physiological processes and plays an important role in the triggering and amplification of the inflammatory response. The interaction of cancer cells with inflammatory cells, in addition to cytokine responses, in the tumor microenvironment may contribute to tumor growth, progression and immunosuppression (28). Therefore, we reasoned that TREM-1 may participate in carcinogenesis *in vivo* mainly through immune-related mechanisms.

Duan et al. (11) showed that TREM-1 induces the expression and secretion of various cytokines and chemokines, thereby promoting the development of the tumor microenvironment and ultimately contributing to tumor progression. These researchers revealed that TREM-1 overexpression upregulates the secretion of IL-1, IL-6, IL-8, MCP-1, MCP-3, MIP-1, TNF-a, GM-CSF, MPO, and VEGF but decreases the secretion of IL-10. We also found positive correlations between TREM-1 and IL-6 (**Figure 3G**), IL-8 (**Figure S2C**, shown as CXCL8), MCP-1(**Figure S2C**, shown as CCL2), the GM-CSF receptor (**Figure S2A**, shown as CSF1R) and IL-10 (**Figure S2A**). In addition, the expression of the TNF-α receptor, TNFRSF1A, was significantly and positively correlated with TREM-1 expression (**Figure 2B**). Immune infiltration is associated with PTC and may play a critical role in carcinogenesis regulation and carcinoma progression (29). Therefore, we hypothesized that in PTC, TREM-1 induces cytokine expression and produces an inflammatory microenvironment. Long-term inflammatory damage induces cell renewal and repair of defective tissues. During the repair process, carcinogens or macrophages can cause DNA damage in cells, and cell proliferation and differentiation become disordered, establishing conditions supporting tumor formation and metastasis.

The metastatic potential of primary tumors can be enhanced *via* exploitation of aberrant immune cell crosstalk (30). Infiltration of immune cells in tumors modulates the local microenvironment and influences cancer progression and the therapeutic response. Our study showed that TREM-1 is significantly correlated with the infiltration of several immune cells (**Figure 3E**). Furthermore, correlation analysis between TREM-1 expression and 28 types of TILs across human cancers revealed that TREM-1 may participate in complicated immune cell crosstalk, possibly explaining why TREM-1 is closely related to lymph node metastasis in PTC.

Triggering receptor expressed on myeloid cells-2 (TREM‐2) is another member of the TREM family. Opposite to TREM-1, TREM-2 functions as an anti‐inflammatory modulator and phagocytic promoter (31). A previous study on melanoma found that the increased ratio of TREM-1 to TREM-2 may promote the pro-inflammatory and pro-tumor state of the tumor microenvironment (32). This evidence suggests a TREM-1/TREM-2 paradigm in which the relative levels of these two TREM family members, instead of the absolute expression levels of one or the other, dictate inflammatory and immune status, which provides a novel approach for TREM-1 functional studies in PTC.

TREM-1 is expressed as a transmembrane receptor complex with the DAP12 chain subunit. TREM-1 signaling activates the RAS gene, activates the mitogen-activated protein kinase (MAPK) pathway and mediates ERK phosphorylation (9). The RAS gene is the most common target for acquired somatic function mutations in human cancer (33). Oncogenic activation of BRAF enhances cancer progression by constitutively promoting RAS-independent MAPK pathway signaling (1). The BRAF V600E mutation is the most prevalent oncogenic mutation in PTC, occurring in an average of 45% of patients (34). As the TREM-1 expression level showed a significant correlation with BRAF mutation and the degree of PTC severity in our study, we assumed that the mechanism underlying this deterioration may be a synergistic interaction between TREM-1 and BRAF *via* MAPK pathway activation.

## Conclusions

The identification of TREM-1 expression alterations in PTC tissues is an important step toward improving our understanding of PTC pathogenesis and identifying new therapeutic and biomarker targets. Several mechanisms have been proposed to explain the possible role of TREM-1 in PTC development. Although the molecular functions of TREM-1 in PTC have been analyzed using bioinformatics approaches, the conclusions have not been confirmed by experiments, and related experimental research is ongoing.

## Data Availability Statement

Publicly available datasets were used in this study. Details can be found in the article.

## Author Contributions

ZX designed the analytical strategies, performed data analyses and wrote the manuscript. XL performed data analysis and wrote the manuscript. YZH, SW, SYW, JS, YCH, and YL performed data analysis. JZ conceived the research and wrote the manuscript. All authors contributed to the article and approved the submitted version.

## Funding

This research was supported by the National Natural Science Foundation of China (81600602).

## Conflict of Interest

The authors declare that the research was conducted in the absence of any commercial or financial relationships that could be construed as a potential conflict of interest.

## Supplementary Material

Supplementary Figure 1 Relationship between TREM-1 expression and gene mutations in PTC. **(A)** Volcano plot of TREM-1 expression and gene mutations in THCA. **(B)** Relationship between TREM-1 expression and *BRAF* mutation.

Supplementary Figure 2 TREM-1 expression is closely related to immunity across human cancers. Correlation analysis between TREM-1 expression and the levels of **(A)** immunoinhibitors, **(B)** MHCs, **(C) c**hemokines and **(D)** receptors across human cancers *via* TISIDB.

Supplementary Table 1 Association between TREM-1 expression and immune cell proportions.

Supplementary Table 2 Association between TREM-1 expression and immune cell abundences.
