# Supplementary figures and images for: Analysis of the Prognostic Value and Potential Molecular Mechanisms of TREM-1 Overexpression in Papillary Thyroid Cancer via Bioinformatics Methods
Source: Front Endocrinol (Lausanne). 2021 May 27;12:646793. doi: 10.3389/fendo.2021.646793 (PMC8190971; doi:10.3389/fendo.2021.646793)

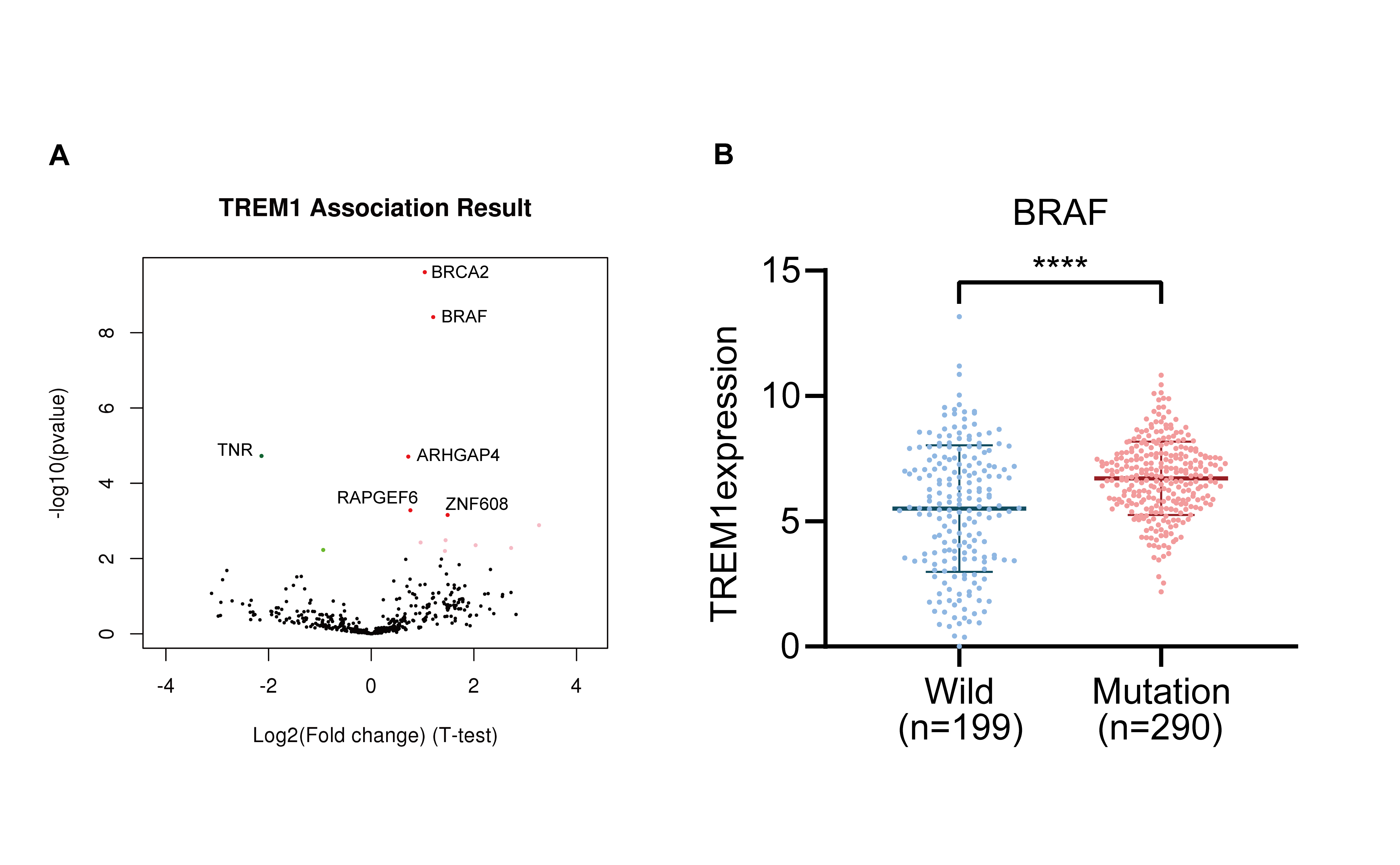

Supplement: Supplementary Figure 1 — Relationship between TREM-1 expression and gene mutations in PTC. (A) Volcano plot of TREM-1 expression and gene mutations in THCA. (B) Relationship between TREM-1 expression and BRAF mutation. [file Image_1.tif]

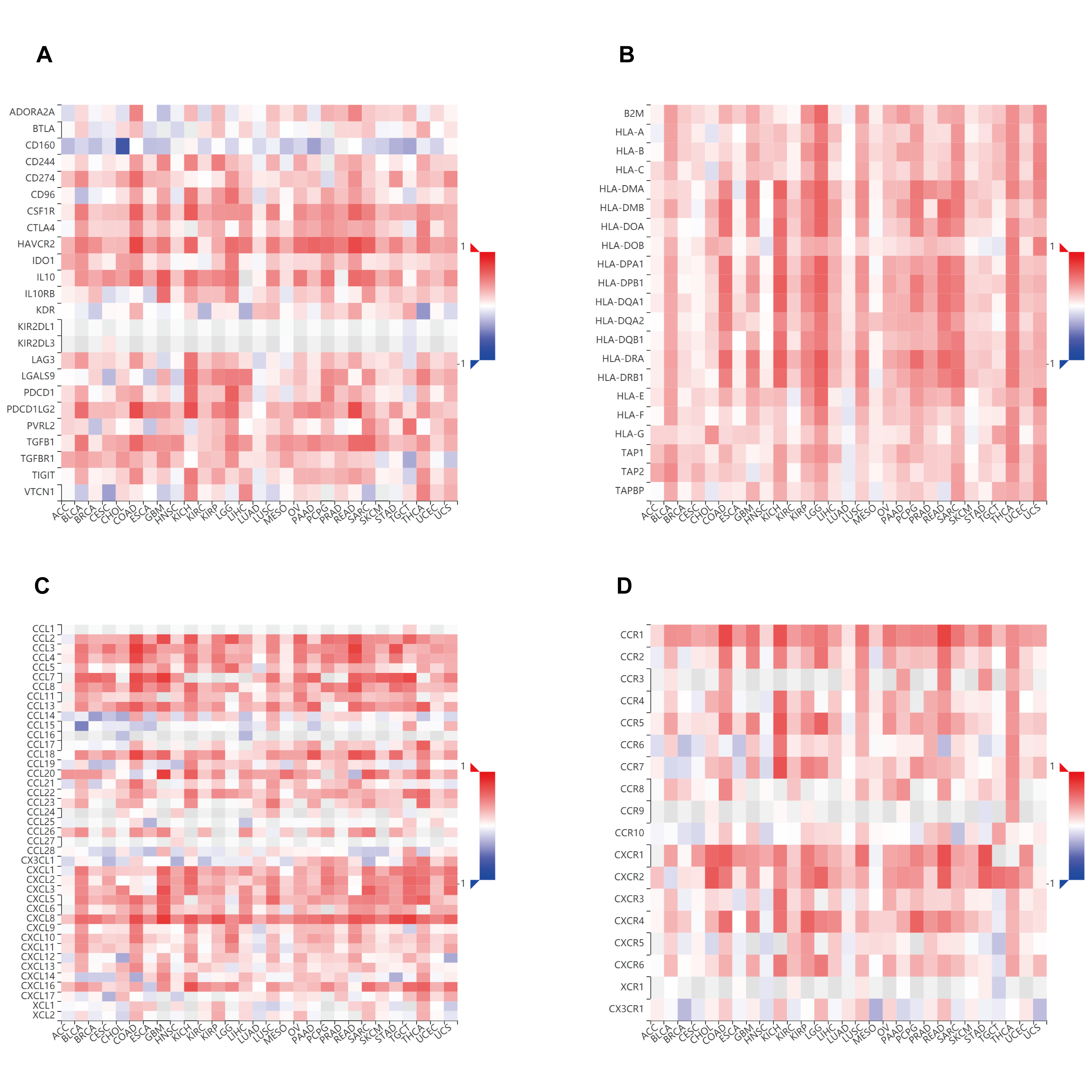

Supplement: Supplementary Figure 2 — TREM-1 expression is closely related to immunity across human cancers. Correlation analysis between TREM-1 expression and the levels of (A) immunoinhibitors, (B) MHCs, (C) chemokines and (D) receptors across human cancers via TISIDB. [file Image_2.tif]
