# Supplementary material for: Analysis of the Prognostic Value and Potential Molecular Mechanisms of TREM-1 Overexpression in Papillary Thyroid Cancer via Bioinformatics Methods
Source: Front Endocrinol (Lausanne). 2021 May 27;12:646793. doi: 10.3389/fendo.2021.646793 (PMC8190971; doi:10.3389/fendo.2021.646793)
Supplement: Supplementary Table 1 — Association between TREM-1 expression and immune cell proportions. [file Table_1.doc]

| Immune Cells | Correlation | *p* value |
| --- | --- | --- |
| Dendritic cells resting | 0.400 | 2.32E-11 |
| Dendritic cells activated | 0.269 | 1.14E-05 |
| Neutrophils | 0.257 | 2.75E-05 |
| Monocytes | 0.251 | 4.35E-05 |
| T cells regulatory (Tregs) | 0.198 | 0.001 |
| T cells CD4 memory resting | 0.082 | 0.189 |
| T cells CD4 memory activated | 0.064 | 0.304 |
| NK cells activated | 0.028 | 0.658 |
| Mast cells activated | 0.018 | 0.770 |
| Mast cells resting | 0.015 | 0.804 |
| Macrophages M0 | 0.014 | 0.822 |
| B cells naive | 0.010 | 0.867 |
| Macrophages M2 | -0.002 | 0.978 |
| Plasma cells | -0.036 | 0.567 |
| Macrophages M1 | -0.069 | 0.271 |
| B cells memory | -0.078 | 0.212 |
| T cells follicular helper | -0.087 | 0.162 |
| Eosinophils | -0.130 | 0.036 |
| T cells CD8 | -0.140 | 0.024 |
| NK cells resting | -0.152 | 0.014 |
| T cells gamma delta | -0.170 | 0.006 |
