# Supplementary material for: Analysis of the Prognostic Value and Potential Molecular Mechanisms of TREM-1 Overexpression in Papillary Thyroid Cancer via Bioinformatics Methods
Source: Front Endocrinol (Lausanne). 2021 May 27;12:646793. doi: 10.3389/fendo.2021.646793 (PMC8190971; doi:10.3389/fendo.2021.646793)
Supplement: Supplementary Table 2 — Association between TREM-1 expression and immune cell abundences. [file Table_2.doc]

| Immune cells | Correlation | *p* value |
| --- | --- | --- |
| Dendritic cells | 0.620 | 1.10E-55 |
| Macrophages | 0.484 | 2.19E-31 |
| Monocytes | 0.457 | 8.46E-28 |
| nTreg | 0.415 | 9.87E-23 |
| iTreg | 0.408 | 5.74E-22 |
| Tr1 | 0.373 | 2.26E-18 |
| Tfh | 0.314 | 3.27E-13 |
| Cytotoxic T cells | 0.304 | 2.05E-12 |
| Exhausted T cells | 0.297 | 6.49E-12 |
| Th1 | 0.270 | 5.57E-10 |
| Th2 | 0.180 | 4.30E-05 |
| Effector memory T cells | 0.153 | 0.0005 |
| CD8 T cells | 0.130 | 0.0031 |
| NK cells | 0.080 | 0.0721 |
| MAIT | 0.001 | 0.9837 |
| NKT | -0.051 | 0.2502 |
| CD4 T cells | -0.148 | 0.0008 |
| B cells | -0.156 | 0.0004 |
| Gamma delta T cells | -0.206 | 2.52E-06 |
| Th17 | -0.212 | 1.25E-06 |
| Central memory T cells | -0.339 | 3.15E-15 |
| CD8 naive T cells | -0.377 | 9.52E-19 |
| Neutrophils | -0.393 | 2.54E-20 |
| CD4 naive T cells | -0.397 | 9.07E-21 |
